# Rehabilitation of Traumatic Acute Subdural Hematoma and Subarachnoid Hemorrhage: A Case Report

**DOI:** 10.7759/cureus.50660

**Published:** 2023-12-17

**Authors:** Amisha P Zade, Shruti S Bhoge, Nikita H Seth, Pratik Phansopkar

**Affiliations:** 1 Musculoskeletal Physiotherapy, Ravi Nair Physiotherapy College, Datta Meghe Institute of Higher Education and Research, Wardha, IND

**Keywords:** case report, physiotherapy, gait impairment, coordination exercises, balance exercises, balance, intra cranial pressure, relaxation techniques, mobility training, acute subdural hematoma

## Abstract

A head injury or cerebrovascular illness may be the cause of acute intracranial hemorrhage. Making a precise diagnosis is challenging since diagnostic imaging might be challenging in both situations. In this case report, an aneurysmal rupture related head injury resulted in an acute subdural hematoma (SHD) after the patient lost consciousness. A 54-year-old male was found in a state of unconsciousness on the ground and was brought to the nearest hospital. Computed tomography (CT) scan showed an oblique fracture involving the bilateral frontal and right parietal bones along with underlying SDH, subarachnoid hemorrhage (SAH), and hemorrhagic contusion along with midline shift. The case report highlights the rehabilitation journey of a patient with acute SDH and SAH. The patient can now sit independently and stand with minimal assistance. Vasospasm detection, prevention, and treatment need to be the norm at that time. This case demonstrates the effectiveness of a comprehensive rehabilitation approach in promoting mobility and independence for patients with traumatic brain injuries.

## Introduction

In acute subdural hematoma (SDH), there is bleeding between the brain and its outermost covering, while subarachnoid hemorrhage (SAH) involves bleeding in the space around the brain. Severe and high-impact injuries usually cause acute SDH. Trauma, cerebral hypotension, and faulty coagulation are further potential causes. Through a combination of functional mobility activities, core stability exercises, balance, gait training, and relaxation exercises, the patient can achieve significant improvement in functional independence; however, numerous risk factors, including advanced age, alcohol consumption, certain medications (antiplatelet, anticoagulants), and, finally, a history of brain damage, can potentially exacerbate this problem [1]. There are comparable risk factors for aneurysmal development in cases of SAH caused by aneurysmal causes [2]. The most frequently noted risk factors include smoking, hypertension, and family history. Alcohol, sympathomimetic medications, and low estrogen levels are further risks. At the dura-arachnoid junction, there is a specialized layer of fibroblasts called the dural border cell layer. It is distinguished by flattened fibroblasts, extracellular gaps, little extracellular collagen, and few cell connections [3]. A tiny portion of instances may not be discovered during the first angiographic examinations, but they may be found after a follow-up angiography and placed in this category [4]. Guidelines on surgical treatment of traumatic SDH have been released by the Foundation for Brain Trauma [5]. Nevertheless, the proportion of SDH patients who can be selected for conservative therapy and the outcomes of these individuals are poorly comprehended. Aneurysmal SAH is an acute neurologic emergency [6]. To stop bleeding again, the aneurysm must be promptly and definitively treated by a craniotomy and clipping or endovascular intervention using coils and/or stents [7]. The study sought to identify risk variables linked to deteriorating and postponed surgery, as well as the percentage of patients who get conservative therapy initially and the percentage of patients who would deteriorate and need surgical evacuation [8]. Ruptures of an aneurysm can cause SAH, which makes up around 5% of all strokes [9]. Frailty in the elderly should be regularly assessed as it may result in increasing morbidity and mortality, opening the door to therapies in the areas of medicine, surgery, nutrition, cognitive function, and physical activity [8]. This study aims to improve understanding of aneurysmal SAH, a rare and serious illness that has a significant risk of severe morbidity and death, in terms of both diagnosis and therapy [10]. This case demonstrates the effectiveness of a comprehensive rehabilitation approach in promoting mobility and independence for patients with traumatic brain injuries.

## Case presentation

**Figure 1:**
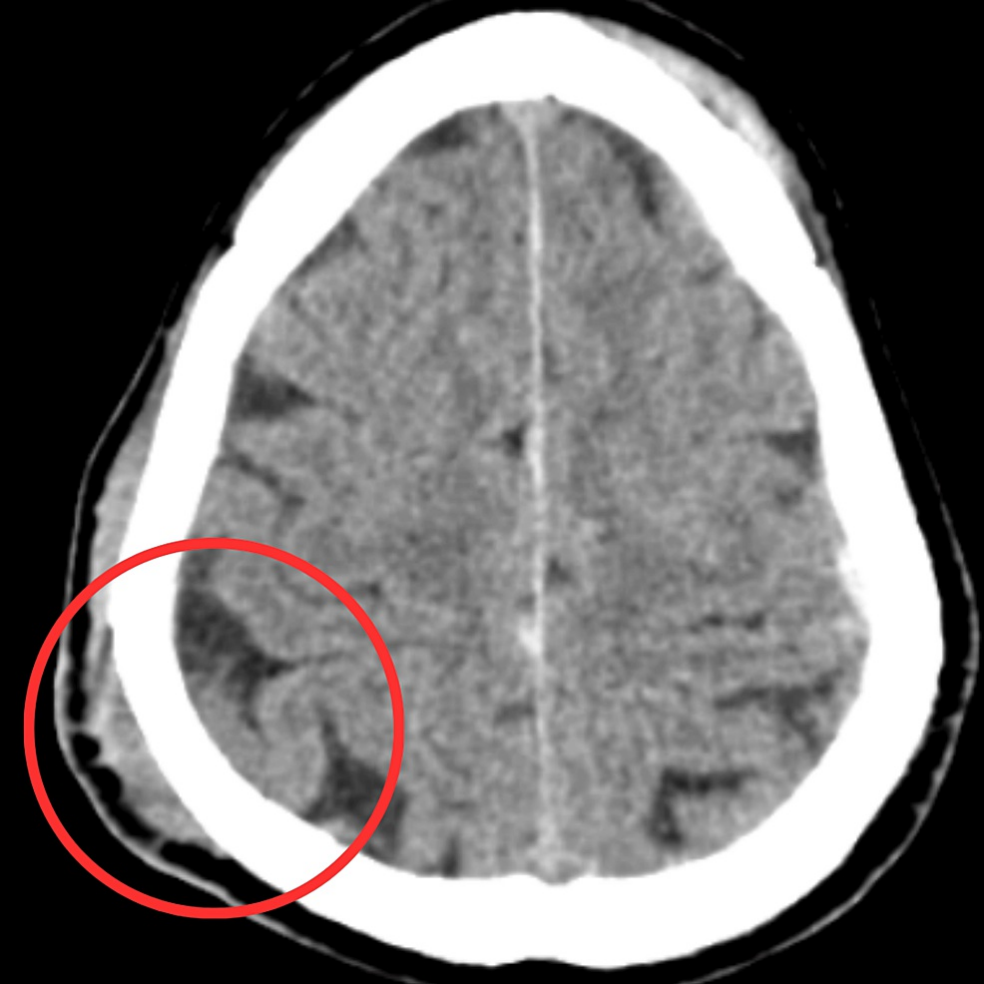
Computed tomography scan of the brain Red circle shows subdural hematoma and subarachnoid hematoma

A 59-year-old male, working as a driver, was brought to Acharya Vinoba Bhave Rural Hospital (AVBRH) in an unconscious state. The patient was alright till October 6, 2023; he was found unconscious on the ground and was brought to the nearest hospital where his Glasgow Coma Scale score was found to be 9. The patient was treated conservatively with medications such as antibiotics, antiemetics, analgesics, antifibrinolytics, and antacids. The patient did not require oxygen support; however, he had undergone certain investigations such as a computed tomography (CT) scan, which showed an oblique fracture involving the bilateral frontal and right parietal bones with underlying SDH, SAH, hemorrhagic contusion, and midline shift, as visible in Figure 1. The patient complained of generalized weakness, headache, and oromotor difficulties. He also revealed no history of alcoholism. He neither gave a record of any comorbidities or bladder or bowel complaints. On October 9, 2023, physiotherapy rehabilitation was commenced with the proper tailor-made protocol for the patient.

Clinical findings

The patient gave his informed permission before the examination started. He appeared cooperative, sluggish, and well-oriented in terms of time, location, and people during assessment. The patient had hemodynamic stability and was afebrile. The patient was seen in a supine lying position with pillows supporting his knee and ankle and his head end raised to a 30° angle. At 19 kg/m^2^, he had a limb ectomorphic body composition. His speech was affected, but his hearing and eyesight were normal. A neurological examination revealed intact senses. Both the lower and upper limbs had less muscular strength and tone. Every deep tendon reflex was attenuated. The Babinski sign was normal. The patient required assistance with basic activities of daily living (ADL). Tables 1, 2 show the findings of reflexes and manual muscle testing, respectively, before starting the rehabilitation.

**Table 1:** Pre-rehabilitation reflex assessment ++, normal reflex; +++, exaggerated reflex; N/A, not applicable

Reflexes	Right	Left
Brachial reflex	N/A	++
Triceps reflex	N/A	++
Knee jerk	++	++
Ankle jerk	+++	+++
Plantar reflex	++	++

**Table 2:** Pre-rehabilitation findings of MMT MMT, manual muscle testing 3: active movement against gravity 4: full range of motion against gravity with minimal resistance N/A: not applicable due to the clavicle fracture

MMT	Right	Left
Shoulder flexors	N/A	3
Shoulder extensors	N/A	3
Abductors of the shoulder	N/A	3
Adductors of the shoulder	N/A	3
Elbow flexors	N/A	4
Elbow extensors	N/A	3
Flexors of the wrist	3	3
Extensors of the wrist	4	4
Flexors of the hip	3	3
Extensors of the hip	4	4
Abductors of the hip	4	4
Adductors of the hip	4	4
Flexors of the knee	3	3
Extensors of the knee	3	3
Ankle dorsiflexors	3	3
Ankle plantar flexors	3	3

Physiotherapy intervention

**Figure 2:**
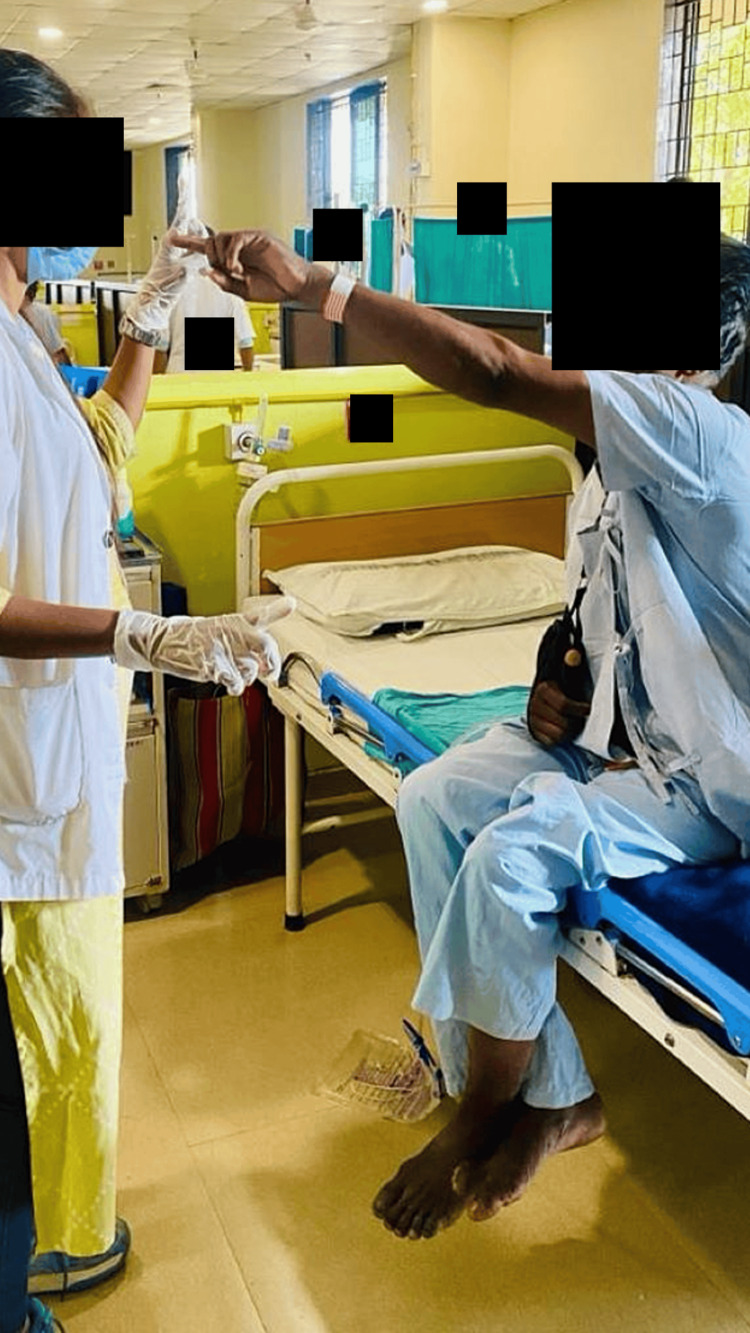
Performing the finger-to-nose test Procedure: The test is performed by alternating between hands, and it helps assess coordination and proprioception. It is commonly used to evaluate motor control and coordination.

**Figure 3:**
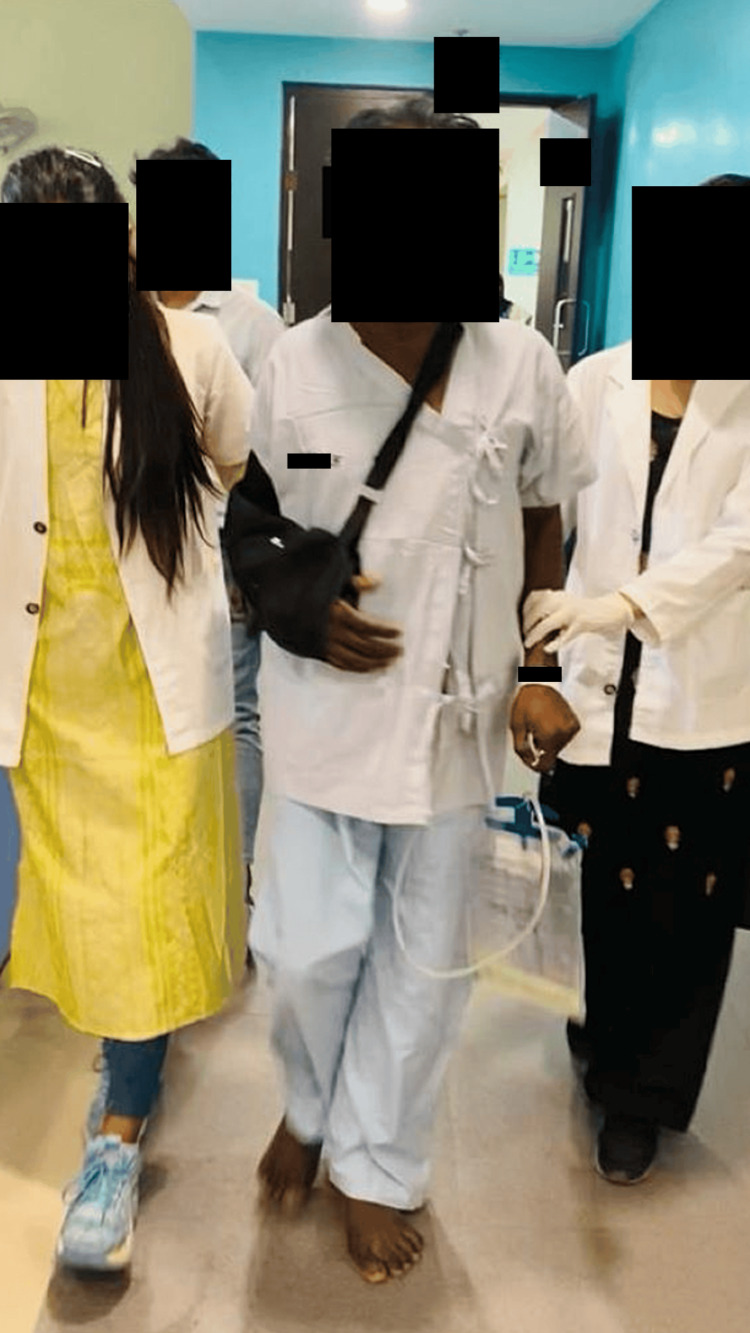
Gait training (weight-shifting and stepping strategies) Gait training helps improve walking ability exercises such as strengthening, balance training, and coordination drills, and also helps the patient regain independence and optimize their walking function.

**Figure 4:**
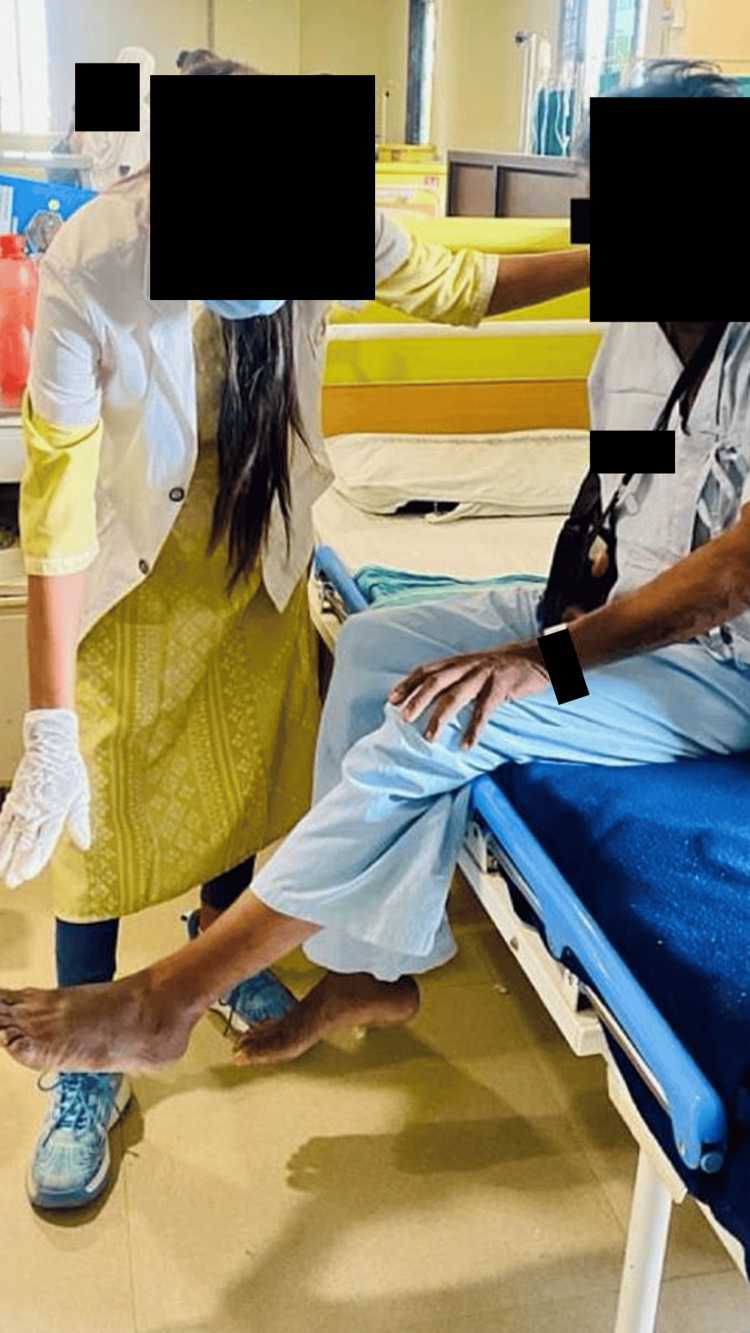
Dynamic quadriceps This focuses on strengthening the leg muscles to improve flexibility and balance.

Physiotherapy rehabilitation protocol was given for four weeks, five times in a week. Physiotherapy intervention received by the patient is summarized in Table 3. The patient was made aware of the condition, and then current physical abilities, including strength, range of motion, balance, and coordination were assessed. To improve their ability to walk and transfers, the patient underwent gait training and bed mobility training, respectively. This training involved practicing with walking assistive devices and learning proper body mechanics. Several factors contribute to deteriorating health problems such as balance impairments, muscle weakness, incoordination, and cognitive dysfunction. The foundation of vestibular rehabilitation is the use of compensatory and adaptive brain processes that are already present in humans. Figures 2-4 show the patient receiving physiotherapy treatment.

**Table 3:** Physiotherapy rehabilitation PNF, proprioceptive neuromuscular facilitation; reps, repetition; N/A, not applicable

Problem identified	Goal	Treatment strategy	Intervention	Progression
Patient and family education	To maintain a positive attitude in the patient toward treatment for early recovery	Interaction of the therapist with the patient and his family	Condition was explained to the patient and his family and they were told about the importance of physiotherapy intervention	Home program was explained
Unable to perform bed mobility independently	To promote mobility	Functional mobility activities	Rolling, weight-shifting	N/A
Inefficient trunk and pelvic control	To improve trunk and pelvic control	Core stability exercise	Pelvic bridging exercises (10 reps with 5 seconds hold, 1 set)	Pelvic muscle strengthening on therapy ball (5 minutes)
Balance and ambulation impairment	To gain static and dynamic balance	Balance and ambulation retraining	PNF gait training (weight-shifting and stepping strategies)	PNF gait training (weight, shifting, and stepping strategies)
Relaxation techniques	Reduce stress, promote calmness, improve well-being	Practice deep breathing, progressive muscle relaxation	Relaxation exercises regularly	Improve ability to achieve relaxation

Functional exercises combined with weight training were found to be useful strategies for increasing patient strength. To increase muscle strength, resistance training using weights and resistance bands was given. Functional exercises served to enhance general strength and coordination by emphasizing motions that are similar to everyday tasks. A safe and customized strength training program for the patient was made. Exercises that target balance, proprioception, and motor control were found to be beneficial in enhancing coordination. Exercises such as walking on uneven terrain, standing on one leg, and using agility ladders or balancing boards might were included in this category. Engaging in regular practice and maintaining a structured routine was found to support cognitive improvement. Table 4 displays the results before and after therapy.

**Table 4:** Results before and after therapy

	Pre-treatment (first day of treatment)	Post-treatment (After four weeks of treatment)
Sitting	Needs mild assistance	Sits independently
Standing	Moderate help is required	Stands without much support
Walking	Moderate help is needed	Minimal assistance needed
Berg balance scale score	16	34
Functional independence measure	Maximum support (patient = 25% or above)	Minimal assistance needed (75% or more of the patient)

## Discussion

SAH and SDH are critical neurological conditions that require comprehensive management strategies, and physiotherapy plays a crucial role in the rehabilitation process. SAH is often caused by the rupture of an aneurysm, while SDH results from bleeding between the brain and its outermost covering. In the acute phase of care, physiotherapists collaborate with the medical team to ensure the safety and stability of the patient. This may involve bedside exercises, gentle range-of-motion activities, and respiratory exercises to prevent complications such as pneumonia or respiratory distress. As the patient progresses, physiotherapy interventions become more intensive, encompassing gait training, balance exercises, and functional activities aimed at restoring independence in daily life. Both conditions can lead to impaired motor function, coordination, and cognitive abilities [11]. Physiotherapy is essential in addressing the physical and functional consequences of SAH and SDH. It focuses on restoring mobility, strength, and balance, and helps patients regain independence in their daily activities. Through tailored exercise programs, physiotherapists work to improve muscle tone, coordination, and flexibility, facilitating the recovery of motor skills compromised by these neurological events [12-14]. Moreover, physiotherapy contributes to the prevention of secondary complications such as muscle atrophy, joint contractures, and respiratory issues, commonly observed in patients recovering from SAH and SDH. Additionally, physiotherapists play a pivotal role in optimizing patients’ overall well-being by addressing pain management and promoting psychological resilience during the rehabilitation process [15-18].

## Conclusions

Based on the case report, the conclusion of the rehabilitation for traumatic acute SDH and SAH is that physical therapy interventions, including exercises, gait training, and non-equilibrium, can effectively improve mobility, strength, and coordination in patients. These interventions should be implemented under a physical therapist’s supervision and tailored to the individual’s specific needs and goals. Additional measures such as pain level, range of motion, functional ability, quality of life, patient satisfaction, and adherence to treatment can be included in the assessment to offer a thorough assessment of the results of the therapy.

## References

[1] Korkmaz Dilmen Ö, Bonhomme V (2023). Management of aneurysmal subarachnoid haemorrhage and its complications: a clinical guide. Turk J Anaesthesiol Reanim.

[2] Cheshire EC, Malcomson RD, Sun P, Mirkes EM, Amoroso JM, Rutty GN (2018). A systematic autopsy survey of human infant bridging veins. Int J Legal Med.

[3] Haines DE, Harkey HL, al-Mefty O (1993). The "subdural" space: a new look at an outdated concept. Neurosurgery.

[4] Pierre L, Kondamudi NP (2023). Subdural hematoma. StatPearls [Internet].

[5] Scholten J, Vasterling JJ, Grimes JB (2017). Traumatic brain injury clinical practice guidelines and best practices from the VA state of the art conference. Brain Inj.

[6] Chung DY, Abdalkader M, Nguyen TN (2021). Aneurysmal subarachnoid hemorrhage. Neurol Clin.

[7] Sharma D (2020). Perioperative management of aneurysmal subarachnoid hemorrhage. Anesthesiology.

[8] Blackwell JN, Keim-Malpass J, Clark MT (2020). Early detection of in-patient deterioration: one prediction model does not fit all. Crit Care Explor.

[9] de Rooij NK, Linn FH, van der Plas JA, Algra A, Rinkel GJ (2007). Incidence of subarachnoid haemorrhage: a systematic review with emphasis on region, age, gender and time trends. J Neurol Neurosurg Psychiatry.

[10] D'Souza S (2015). Aneurysmal subarachnoid hemorrhage. J Neurosurg Anesthesiol.

[11] Salles JI, Velasques B, Cossich V, Nicoliche E, Ribeiro P, Amaral MV, Motta G (2015). Strength training and shoulder proprioception. J Athl Train.

[12] Konrad HR, Tomlinson D, Stockwell CW, Norré M, Horak FB, Shepard NT, Herdman SJ (1992). Rehabilitation therapy for patients with disequilibrium and balance disorders. Otolaryngol Head Neck Surg.

[13] Schoenfeld BJ, Grgic J, Krieger J (2019). How many times per week should a muscle be trained to maximize muscle hypertrophy? A systematic review and meta-analysis of studies examining the effects of resistance training frequency. J Sports Sci.

[14] Prasertsakul T, Kaimuk P, Chinjenpradit W, Limroongreungrat W, Charoensuk W (2018). The effect of virtual reality-based balance training on motor learning and postural control in healthy adults: a randomized preliminary study. Biomed Eng Online.

[15] Lim JH, Lee HS, Song CS (2021). Home-based rehabilitation programs on postural balance, walking, and quality of life in patients with stroke: a single-blind, randomized controlled trial. Medicine (Baltimore).

[16] Abouhashem S, Eldawoody H (2022). Functional outcome after primary decompressive craniectomy for acute subdural hematoma in severe traumatic brain injury. Turk Neurosurg.

[17] Modi NJ, Agrawal M, Sinha VD (2016). Post-traumatic subarachnoid hemorrhage: a review. Neurol India.

[18] Bajsarowicz P, Prakash I, Lamoureux J, Saluja RS, Feyz M, Maleki M, Marcoux J (2015). Nonsurgical acute traumatic subdural hematoma: what is the risk?. J Neurosurg.

